# Diets High in Heat-Treated Soybean Meal Reduce the Histamine-Induced Epithelial Response in the Colon of Weaned Piglets and Increase Epithelial Catabolism of Histamine

**DOI:** 10.1371/journal.pone.0080612

**Published:** 2013-11-19

**Authors:** Susan Kröger, Robert Pieper, Hubert G. Schwelberger, Jing Wang, Carmen Villodre Tudela, Jörg R. Aschenbach, Andrew G. Van Kessel, Jürgen Zentek

**Affiliations:** 1 Institute of Animal Nutrition, Department of Veterinary Medicine, Freie Universität Berlin, Berlin, Germany; 2 Molecular Biology Laboratory, Department of Visceral, Transplantation and Thoracic Surgery, Medical University, Innsbruck, Innsbruck, Austria; 3 Department of Animal and Poultry Science, University of Saskatchewan, Saskatoon, Canada; 4 Department of Animal Production, Faculty of Veterinary Science, University of Murcia, Murcia, Spain; 5 Institute of Veterinary Physiology, Department of Veterinary Medicine, Freie Universität Berlin, Berlin, Germany; National Institute of Agronomic Research, France

## Abstract

We examined the influence of dietary fermentable protein (fCP) and fermentable carbohydrates (fCHO) on the colonic epithelial response to histamine in pigs. Thirty-two weaned piglets were fed 4 diets in a 2 × 2 factorial design with low fCP/low fCHO, low fCP/high fCHO, high fCP/low fCHO and high fCP/high fCHO. After 21-23 days, the pigs were killed and tissue from the proximal colon was stimulated with carbachol, histamine, PGE_2_ or sodium hydrogen sulphide in Ussing chambers. Changes in short-circuit current and tissue conductance were measured. Diamine oxidase, histamine *N*-methyltransferase, stem cell growth factor receptor, Fc-epsilon receptor I and cystic fibrosis transmembrane conductance regulator gene expression was determined. Activities of diamine oxidase and histamine *N*-methyltransferase and numbers of colonic mast cells were measured. The change in the short-circuit current in response to histamine was lower (*P* = 0.002) and tended to be lower for PGE_2_ (*P* = 0.053) in high fCP groups compared to low fCP groups, irrespective of fCHO. Additionally, the change in tissue conductance after the application of histamine was lower (*P* = 0.005) in the high fCP groups. The expression of histamine *N*-methyltransferase mRNA (*P* = 0.033) and the activities of diamine oxidase (*P* = 0.001) and histamine *N*-methyltransferase (*P* = 0.006) were higher with high fCP in comparison with low fCP. The expression of mast cell markers, stem cell growth factor receptor (*P* = 0.005) and Fc-epsilon receptor I (*P* = 0.049) was higher with high fCP diets compared to diets low in fCP, whereas the mast cell count did not differ between groups. The expression of the cystic fibrosis transmembrane conductance regulator was reduced (*P* = 0.001) with high fCP diets compared to low fCP diets. The lower epithelial response to histamine and PGE_2_ and elevated epithelial histamine inactivation suggests an adaptation to high fCP diets.

## Introduction

Post-weaning diarrhoea (PWD) is a serious digestive disorder in piglets and is often caused by intestinal hypersecretion of chloride, followed by water efflux into the gut lumen. In the weanling pig, enterotoxigenic strains of *Escherichia coli* are recognized as causative. The dietary protein concentration is considered to be an important factor in the development of PWD [1,2]. Feeding diets with low protein content decreased PWD in weaned pigs compared to diets rich in protein [3]. This was also the case when the pigs were challenged with *E. coli* [4]. Protein-derived metabolites may induce inflammatory reactions in the intestinal epithelium or promote chloride secretions into the gut lumen. Fermentable carbohydrates are considered to prevent negative effects of protein fermentation in the gut. For example, the addition of dietary fibre to the diet decreased *E. coli* counts and reduced the adhesion of *E. coli* to the intestinal mucosa [5,6]. There are also some indications, that an addition of mostly insoluble dietary fibre may decrease PWD caused by an imbalance of proteins and carbohydrates entering the large bowel of newly weaned piglets [7]. We have previously shown that diets high in fermentable protein affected the colonic expression of cytokines, mucus genes and oxidative stress parameters independently of the presence of dietary fermentable carbohydrates [8]. Reasons are as yet unknown and information on the influence of these effects on the intestinal barrier function remains scarce. Intestinal infections or inflammatory processes are associated with higher luminal concentrations of prostaglandin E_2_ (PGE_2_) [9]. PGE_2_ acts pro-inflammatory, mediates intestinal chloride secretion and augments recovery of the intestinal barrier function [10,11]. Diets high in fermentable protein are also associated with an increase of potentially toxic metabolites such as biogenic amines, ammonia, volatile phenols, indoles or gaseous metabolites such as hydrogen sulphide [12–14]. Some of these compounds, namely histamine and hydrogen sulphide, are known to induce chloride secretion into the gut lumen [15–19]. Histamine can originate from both, luminal microbial decarboxylation of histidine as well as from mast cells and some other cells within the epithelium [20]. Mechanisms for the intestinal inactivation of histamine in colonic tissues have been described including the degrading enzymes diamine oxidase (DAO) and histamine N-methyltransferase (HMT) [21–23].

The current study hypothesized that diets high in fermentable protein may increase the secretory response of the porcine large intestinal epithelium, and that metabolites or substances such as histamine, sodium hydrogen sulphite (NaHS) and prostaglandin E_2_ (PGE_2_) could be a primary source for the frequently observed changes in faecal consistency that are associated with high protein diets in pigs. These effects should be ameliorated with the addition of fermentable carbohydrates. The data, however, show that the histamine- and PGE_2_-induced secretory response was reduced by diets high in fermentable protein, irrespective of fermentable carbohydrates, and that several factors such as histamine bioelimination and intestinal histamine concentrations may be involved in this reaction.

## Materials and Methods

### Animals, housing and diets

The institutional and national guidelines for the care and use of animals were followed and the study was approved by the State Office of Health and Social Affairs ‘Landesamt für Gesundheit und Soziales Berlin’ (LAGeSo Reg. Nr. 0249/10).

A total of 32 weaning piglets (Euroc x Piétrain; 8.0 ± 1.6 kg initial body weight) from 8 litters (2 male and 2 female each) were assigned to one of 4 treatment groups in a 2 × 2 factorial design. Piglets were housed in pairs in commercial flat-deck pens. Water and feed were provided *ad libitum*. The room temperature was maintained at 26°C on the day of weaning and reduced at regular intervals and maintained at 22°C during the period of 20-23 days post weaning. The lighting program was set to 16 h light and 8 h dark, with the lights being switched on at 4 am. The diets were based on corn, wheat and soybean meal, and formulated to meet or exceed the requirements of weaning piglets. The specific dietary treatments were: low fCP/low fCHO (14.5% Crude Protein, CP/14.5% Total Dietary Fibre, TDF); low fCP/high fCHO (14.8% CP/16.6% TDF); high fCP/low fCHO (19.8% CP/14.5% TDF) and high fCP/high fCHO (20.1% CP/18.0% TDF) as indicated in Table S1. The high fermentable protein diets were formulated by supplementing the basal diet with steam autoclaved (124°C for 20 min) soybean meal at the expense of wheat. The fermentable carbohydrates were supplied as wheat bran and sugar beet pulp to replace corn and wheat as described previously [8].

Additionally, 6 piglets at the age of 38 ± 1 days, that received commercial standard diets were used for functional electrophysiological analyses. 

### Sampling and tissue preparation

Pigs (n=8/day) from each treatment were killed on the experimental days 20-23. The piglets were fed their morning meal such that euthanasia occurred 4.5 (+/- 15 min) hours after the meal. The piglets were anesthetized with 20 mg/kg BW of ketamine hydrochloride (Ursotamin®, Serumwerk Bernburg AG, Germany) and 2 mg/kg BW of azaperone (Stresnil®, Jansen-Cilag, Germany) prior to euthanasia with an intracardial injection of 10 mg/kg BW of tetracaine hydrochloride, mebezonium iodide and embutramide (T61®, Intervet, Germany). Following euthanasia, the entire gastrointestinal tract was removed and tissue of the proximal colon was sampled and immediately used for electrophysiological measurements. Additional samples were taken and either snap frozen in liquid nitrogen to determine the gene expression of *DAO*, *HMT*, stem cell growth factor receptor (*C-Kit*), Fc-epsilon receptor I (*FcεRI*) and cystic fibrosis transmembrane conductance regulator (*CFTR*) and for the measurement of enzyme activities of DAO and HMT. All mentioned enzymes and receptors are related to histamine. Either they are histamine-degrading enzymes (DAO and HMT) or they are marker of histamine-releasing mast cell (C-Kit and FcεRI). The CFTR is a chloride transporting ion channel, which is known to be involved in the epithelial response to histamine in the proximal colon. Bouin-fixed tissues were used for immunohistochemical staining. 

### Electrophysiological measurements

For determining colonic electrophysiological parameters, the intestinal segments were immediately placed in an oxygenated (95% O_2_/5% CO_2_) and pre-warmed modified Krebs-Ringer buffer solution (pH adjusted to 7.4, containing in mmol per litre: NaCl, 115; KCl, 5; CaCl_2_, 1.5; MgCl_2_, 1.2; NaH_2_PO_4_, 0.6; Na_2_HPO_4_, 2.4; NaHCO_3_, 25; glucose, 10; and mannitol, 2). The epithelium was stripped of the serosal and muscle layers and immediately mounted in Ussing chambers with an exposed area of 1.31 cm^2^. The apical and basolateral side of the tissue were bathed in 15 ml of the buffer solution at 38°C through surrounding water-jacketed reservoirs. Continuous gassing with carbogen was provided. Electrical measurements were obtained by a microcomputer-controlled voltage/current clamp (K. Mussler Scientific Instruments, Germany). The transepithelial potential difference in response to bipolar 50 μA current pulses generated for 200 ms and the tissue conductance (Gt) was calculated every 6 sec by Ohm’s law. After equilibration for approximately 15-30 min, tissues were short-circuited by clamping the voltage at 0 mV. After reaching the baseline, chloride ion secretion was determined after the addition of either carbamylcholine chloride (carbachol; final concentration in the chamber: 100 µmol/l) or PGE_2_ (final concentration: 20 µmol/l) to the serosal side of 2 chambers per pig. After 30 min histamine hydrochloride (final histamine concentration: 100 µmol/l) was added to the serosal side of the 2 chambers which were previously treated with carbachol. While the other three secretagogues were applied to the serosal compartment, NaHS (final concentration: 1 mmol/l), a H_2_S donor, was added to the mucosal side of the chambers previously treated with PGE_2_. Earlier investigations showed similar responses after mucosal and serosal administration [19]. Two chambers served as controls. The baseline of short-circuit current (Isc) and Gt was measured by calculating the mean of the last 3 min before the secretagogues were added. The change of Isc (ΔIsc) and Gt (ΔGt) was determined for all four substances by subtracting the peak Isc/Gt after 3 min from the basal Isc/Gt as indirect measure of the electrolyte transport. 

Tissue from the additional 6 control piglets was used for the determination of the interaction between the epithelial sodium channel (ENaC) and histamine in the pig colon, as electrophysiological net responses might be related to the activity of ENaC channels. Therefore, amiloride (final concentration: 1 mmol/l) was added to the apical side in half of the chambers 15 min before the addition of histamine (final concentration: 100 µmol/l) to the serosal side in all chambers.

### RNA extraction and gene expression analysis

The colon tissue was ground into fine powder over liquid nitrogen and 50 mg were used for extraction of the total RNA using the NucleoSpin® RNAII kit (Macherey-Nagel GmbH & Co. KG, Germany). The expression of *DAO* and *HMT*, as indicators for the histamine metabolism, *C-Kit* and *FcεRI*, as marker molecules expressed by mast cells and *CFTR* as a chloride transporting ion channel were determined, and 18S rRNA, 60S ribosomal protein L19 (*RPL19*), hypoxanthine phosphoribosyltransferase I (*HPRTI*) and β-actin were used as housekeeping genes for data normalization. Primers (Table S2) were designed based on published sequences of the above mentioned target genes in the pig using the NCBI online primer design tool. Quantitative real-time PCR was performed using the one-step QRT-PCR master mix kit (Brilliant®II SYBR®Green, Agilent Technologies, USA) as described previously [8]. The obtained Ct values were normalized and arbitrary values were calculated and used for statistical comparisons.

### Determination of enzyme activities of DAO and HMT

For determination of DAO and HMT activities, frozen tissue samples (50 mg) were homogenised with a 5 mm steel bead in a TissueLyser II instrument (Qiagen, Germany) for 5 min at 30 Hz in 1 ml of 20 mM bis-Tris hydrochloride pH 7.0 containing 5 mM dithiothreitol and 1 x Complete Protease Inhibitor Cocktail (Roche, Germany). The homogenates were cleared by centrifugation for 10 min at 23,000 x *g* and the supernatants were used to determine DAO and HMT activities as well as protein concentrations.

DAO activity was determined in a total volume of 100 µl using 20 µl of the homogenate, employing a radiometric procedure with [1,4-^14^C]putrescine hydrochloride (0.45 mM, specific radioactivity 8.214 GBq/mol; Amersham Pharmacia Biotech, UK) as the substrate for 30 min at 37°C as described previously [24]. The background activity was measured in assays without homogenate.

HMT activity was measured in a total volume of 100 µl using 20 µl of the homogenate by transmethylation of histamine by S-adenosyl-L-[methyl-^14^C]methionine (50 µM, specific radioactivity 74 GBq/mol (Amersham Pharmacia Biotech, UK) for 30 min at 37°C [25]. The background activity was measured in assays without histamine.

The protein concentration of the homogenates was determined by the Bradford method [26], using a commercially available kit (Bio-Rad Laboratories, Germany). Mean specific enzymatic activities from duplicate assays for each sample were calculated in microunits per mg protein (µU/mg), where 1 µU converts 1 pmol substrate per minute at 37°C.

### Immunohistochemical staining of mast cells

Tissue from the proximal colon were cut open along the mesenterial line, rinsed carefully with PBS, fixed on cork plates and placed in Bouin’s solution. After 76 h the samples were rinsed and stored in 70% ethanol followed by 80% ethanol. The samples were embedded in paraffin wax (Paraplast plus) according to standard protocols [27]. Sections of 5 µm were cut from the paraffin blocks by using a rotary microtome. Mast cells were stained according to a modified protocol by Zielschot [28]. After dewaxing and rehydration, endogenous peroxidase was blocked for 30 min in hydrogen peroxide (0.5%), followed by antigen demasking in citrate buffer at 90°C for 20 min. Tissue sections were blocked with 1:5 diluted inactivated normal goat serum (Dako Deutschland GmbH, Germany), prior to overnight incubation at 4°C with a polyclonal rabbit anti-human CD117 (C-Kit) antibody (Dako Deutschland GmbH, Germany) diluted in 1% bovine serum albumin (1:750). Afterwards, the slides were incubated for 30 min with biotinylated goat anti-rabbit secondary antibody (Biologo, Germany) diluted in phosphate buffer (1:200) prior to a 30 min incubation with avidin-biotin-peroxidase complex (ABC-reagent; Vectastain® Elite ABC Kit, Vector Laborities Inc., USA; purchased from Biologo, Germany) to amplify the signal of the primary antibody. After 10 min in a 1:50 dilution of biotinyl tyramide (TSA Biotin Kit, PerkinElmer, Inc., USA), the slides were incubated for another 15 min in ABC-reagent, followed by an enzyme histochemical reaction with 3´3 Diaminobenzidin-tetrahydrochloride for 10 min and counterstaining with Mayer´s hemalaun for 30 s.

From each section (n = 4 per animal), three non-overlapping areas of the *Lamina submucosa* (incl. *L. muscularis mucosae*) and the *Lamina propria* were defined, and cells were counted by one blinded examiner to estimate the mast cell number. The mean was provided in cells/mm^2^. The examination was performed by using a microscope (Zeiss Photomicroscope, Germany) that included a digital camera (Olympus DP72, Japan) and by using cellSens Standard software (Olympus, 2010).

### Statistical analysis

Data were analysed using generalized linear model procedures (GLM) in SPSS (version 18.0, Chicago, IL, USA) with fCP and fCHO and their interaction as sources of variation. Two-sample t-Test was used for analysing differences in the histamine response with or without pre-treatment with amiloride after the data were determined as normally distributed by the Shapiro-Wilk-Test. Differences at *P* < 0.05 were considered significant. All data were presented as mean ± standard error (SE).

## Results

### Baseline values for colonic short-circuit current and tissue conductance

The baselines were comparable between the feeding groups (Table S3). Only the basal Isc of PGE_2_ chambers indicated an interaction between fCP and fCHO (Table S3).

### Secretagogue-induced colonic chloride secretion

The addition of 100 µmol/l histamine to the serosal side of the colonic tissue resulted in a rapid change of Isc in all feeding groups which peaked after 3 min. The ΔIsc after 3 min was lower (*P* < 0.05) in the groups receiving the diets with high concentrations of fCP with no apparent influence of fCHO (Table 1). Correspondingly, the ΔIsc tended to be lower (*P* = 0.053) after the addition of PGE_2_ in groups receiving high fCP/low fCHO and high fCP/high fCHO (Table 1). The addition of carbachol or NaHS resulted in an increase of Isc without group effects (Table 1). 

**Table 1:** Change of short circuit current (µA/cm^2^) in the colonic tissue 3 min after the application of carbachol, PGE_2_, histamine (all basolateral) and NaHS (apical) of piglets fed diets containing low or high concentration of fCP or fCHO (data are presented as means ± SE, *n* = 8).

	low fCP		high fCP		*P* values		
	low fCHO	high fCHO	low fCHO	high fCHO	fCP	fCHO	fCP x fCHO
Carbachol	28.9 ± 4.1	28.6 ± 6.0	30.3 ± 13	32.9 ± 2.7	0.66	0.87	0.82
PGE_2_	9.93 ± 2.6	8.89 ± 2.1	5.75 ± 3.5	6.42 ± 1.6	0.05	0.68	0.63
Histamine	30.0 ± 3.9	19.2 ± 5.3	10.2 ± 4.7	13.5 ± 3.2	0.002	0.37	0.09
NaHS	4.00 ± 0.7	3.00 ± 1.0	5.25 ± 2.1	4.08 ± 1.3	0.31	0.34	0.94

PGE_2_, prostaglandin E_2_; NaHS, sodium hydrogen sulphite; fCP, fermentable crude protein; fCHO, fermentable carbohydrates

The ΔIsc after the serosal addition of histamine did not differ when the tissue was pre-treated without (89.0 ± 8.47 µA/cm^2^) or with amiloride (107.4 ± 9.61 µA/cm^2^) (*P* > 0.05).

### Tissue conductance after application of secretagogues

Serosal addition of histamine increased ΔGt (*P* < 0.05) in the high fCP groups compared to the low fCP groups irrespective of the fCHO addition (Table 2). The ΔGt did not differ between the groups after the application of carbachol, PGE_2_ and NaHS (Table 2). 

**Table 2:** Change of colonic tissue conductance (mS/cm^2^) 3 min after the application of carbachol, PGE_2_, histamine (all basolateral) and NaHS (apical) of piglets fed diets containing low or high concentration of fCP or fCHO (data are presented as means ± SE, *n* = 8).

	low fCP		high fCP		*P* values		
	low fCHO	high fCHO	low fCHO	high fCHO	fCP	fCHO	fCP x fCHO
Carbachol	1.13 ± 0.9	0.56 ± 0.5	-0.20 ± 0.6	0.82 ± 0.3	0.35	0.70	0.17
PGE_2_	-0.29 ± 0.5	0.21 ± 0.2	-0.52 ± 0.2	-0.52 ± 0.4	0.13	0.43	0.43
Histamine	1.77 ± 0.6	0.89 ± 0.3	0.20 ± 0.2	0.50 ± 0.1	0.005	0.40	0.09
NaHS	0.44 ± 0.2	0.53 ± 0.3	0.50 ± 0.2	0.30 ± 0.1	0.78	0.88	0.40

PGE_2_, prostaglandin E_2_; NaHS, sodium hydrogen sulphite; fCP, fermentable crude protein; fCHO, fermentable carbohydrates

### Gene expression of DAO, HMT, C-Kit, FcεRI and CFTR in the colon tissue

The gene expression of *HMT* was higher (*P* < 0.05) in the proximal colon tissue of weaning piglets fed with diets containing high amounts of fCP (Table 3). In contrast, the *DAO* expression was not significantly altered by dietary treatments. The inclusion of fCHO did not affect the expression of the two enzymes.

**Table 3:** Gene expression (arbitrary values) of *DAO*, *HMT*, *C-Kit*, *FcεRI* and *CFTR*, and enzyme activity (µU/mg protein) of DAO and HMT in the colonic tissue of piglets fed diets containing low or high concentration of fCP or fCHO (data are presented as means ± SE, *n* = 8).

	low fCP		high fCP		*P* values		
	low fCHO	high fCHO	low fCHO	high fCHO	fCP	fCHO	fCP x fCHO
Gene expression^1^							
*DAO*	0.97 ± 0.1	1.05 ± 0.1	1.19 ± 0.3	1.02 ± 0.2	0.55	0.77	0.42
*HMT*	0.88 ± 0.1	0.84 ± 0.1	1.05 ± 0.1	0.96 ± 0.1	0.033	0.35	0.70
*C-Kit*	0.46 ± 0.1	0.37 ± 0.1	0.63 ± 0.1	0.56 ± 0.1	0.005	0.22	0.82
*FcεRI*	0.43 ± 0.2	0.71 ± 0.1	0.99 ± 0.3	0.95 ± 0.1	0.049	0.30	0.58
*CFTR*	0.63 ± 0.06	0.69 ± 0.07	0.44 ± 0.03	0.49 ± 0.03	<0.001	0.22	0.96
Enzyme activity^2^							
DAO	40.8 ± 5.3	43.4 ± 5.1	77.6 ± 14	64.4 ± 13	0.001	0.55	0.37
HMT	290 ± 32	286 ± 38	376 ± 56	394 ± 33	0.006	0.84	0.77

DAO, diamine oxidase; HMT, histamine *N*-methyltransferase; C-Kit, stem cell growth factor receptor; *FcεRI*, Fc epsilon receptor I; CFTR, cystic fibrosis transmembrane conductance regulator; fCP, fermentable crude protein; fCHO, fermentable carbohydrates

^1^ Arbitrary values

^2^ µU/mg protein

The expression of *C-Kit* and *FcεRI* was higher (*P* < 0.05) in the tissue of piglets fed the diets high in fCP compared to the low fCP groups (Table 3). Both were not affected by higher levels of fCHO.

The expression of *CFTR* was diminished (*P* < 0.05) in the tissue of the proximal colon from piglets receiving the high fCP diets (Table 3).

### Enzyme activities of DAO and HMT in the colonic tissue

 The activities of both enzymes, DAO and HMT, were increased (*P* < 0.05) in the groups fed the high fCP diets, independent of the fCHO content (Table 3). 

### Immunohistochemistry

The distribution of mast cells in the *Lamina propria* and in the *Lamina submucosa* of the proximal colon did not differ between the groups (Table 4), whereby the number of mast cells was higher in the *L. submucosa* compared to the *L. propria* (Table 4). 

**Table 4:** Mast cell count (cells/mm^2^) in the Lamina propria (LP) and in the Lamina submucosa (LS; including *Lamina muscularis mucosae*) of the proximal colon (data are presented in mean ± SE).

	low fCP		high fCP		*P* values		
	low fCHO	high fCHO	low fCHO	high fCHO	fCP	fCHO	fCP x fCHO
LP	17.9 ± 10.6	12.5 ± 5.0	27.6 ± 12.6	20.1 ± 8.1	0.22	0.54	0.921
LS	87.9 ± 36.2	159.4 ± 186	116.2 ± 122	148.6 ± 161	0.70	0.21	0.88

LP, *Lamina propria*; LS, *Lamina submucosa*; fCP, fermentable crude protein; fCHO, fermentable carbohydrates

## Discussion

Diets with higher protein levels can lead to an elevated bacterial protein catabolism with the formation of potentially harmful biogenic amines such as histamine, tyramine, putrescine and cadaverine in pigs [29]. As reported previously, piglets from the present study fed diets high in fermentable protein had increased branched chain fatty acids, ammonia, putrescine, histamine and spermidine concentrations in the colonic digesta [8]. Furthermore, host response measures indicated upregulated inflammatory responses and cell turnover in the colon epithelium [8]. Interestingly piglets in the high fCP/low fCHO group had lower faecal scores compared to the piglets from the other groups indicating more watery faeces [8]. This may reflect a negative dietary effect on the absorptive capacity for electrolytes in the hindgut or increased chloride secretion under these conditions. It was therefore of interest to relate those *in vivo* findings to *in vitro* data using colonic tissue from the different dietary groups. The basal values for Isc and Gt however suggest more or less stable tissue conditions with undisturbed balance of secretion, absorption and permeability for electrolytes, mainly sodium, chloride and bicarbonate [30,31]. It is assumed that the change of Isc in response to histamine is mainly due to chloride secretion. The serosal histamine application to the pig proximal colon increased the serosal-to-mucosal chloride flux, maintained the mucosal-to-serosal flux, and therefore decreased the chloride net absorption [17]. Additionally, in the porcine distal colon, the serosal-to-mucosal sodium flux was increased and the sodium net absorption decreased [16]. Almost three-quarters of the histamine response seemed to be chloride-dependent and just one-quarter HCO_3_^-^-dependent, at least in the distal colon [16]. Referring to these data, an involvement of HCO_3_^-^ in the histamine response of the proximal colon cannot be totally excluded. However, the influence of epithelial sodium channels in the current study can be neglected, as blocking of ENaC with amiloride did not reduce the tissue histamine response. 

Histamine is one of the physiologically most interesting biogenic amines produced from L-histidine in the gastrointestinal digesta and released from mast cells in the intestinal mucosa. Although little research has been conducted to date on that aspect, many gut bacteria can be potential histamine producers. The microbial degradation of the amino acid histidine requires the enzyme L-histidine decarboxylase [20]. *Enterobacteriaceae* are considered to be the largest and most diverse group of histamine producers in food and in the intestinal tract [32,33], but strains of *Clostridium perfringens* [34] and many lactobacilli [35] have also been identified as producers of L-histidine decarboxylase. Interestingly, the application of histamine to the serosal compartments of the Ussing chambers provoked increases of ΔIsc and ΔGt, which were both significantly lower in the piglets of the high fCP groups. This is remarkable and somewhat unexpected, as PWD and scours in weaned pigs are more frequently observed with high protein diets [36,37]. Although histamine is mainly known for its pro-inflammatory effects, it may, under certain conditions, also have anti-inflammatory and immunoregulatory functions. For example, a probiotic strain of *L. reuteri* with known immunomodulatory effects in the gastrointestinal tract was identified as a producer of histamine, which suppressed the production of the pro-inflammatory tumor necrosis factor [38]. 

The application of carbachol and NaHS provoked an increase in the ΔIsc without any differences between the feeding groups. PGE_2_ caused a numerically lower increase of ΔIsc in the high fCP groups. The results of histamine and PGE_2_-induced chloride secretion compared to carbachol and NaHS-induced chloride secretion indicate different pathways of transepithelial chloride secretory mechanisms. Histamine was reported to bind mainly to H_2_-receptors in the proximal colon of piglets [17]. These receptors, as well as PGE_2_, are known to act predominantly via the cAMP pathway [39,40], which leads to an activation of the apical chloride channel CFTR. On the other hand, the basolaterally added neurotransmitter carbachol results in an elevation of intracellular calcium concentrations, which activates potassium channels and calcium-activated chloride channels [40]. Hydrogen sulphide increased cytoplasmic calcium concentrations in the rat colon and stimulated colonic ion secretion by apical as well as by basolateral epithelial potassium channels [19]. These results suggest a specific down-regulation of the cAMP/CFTR pathway in addition to altered histamine catabolism when feeding high fCP diets. The latter hypothesis was substantiated by analysing the expression of the epithelial CFTR in the present study. The identified decreased abundance of *CFTR* in the high fCP groups can plausibly explain the decreased Isc response to histamine and, as a trend, also to PGE_2_. 

It is not clear whether and to which extent luminal vs. epithelial histamine have an impact on tissue function and the secretory processes and how this depends on an interplay between these two histamine levels. In the colon, the luminal histamine concentrations can be considered as completely microbially derived. The dietary intake of histamine through elevated feed levels can be most likely excluded in this study, because the ingredients of the diets did not typically contain pre-formed histamine [41]. 

Increased luminal histamine levels in the colon digesta of high fCP-fed pigs were described in our previous report [8]. The tissue samples showed an increased expression of *C-Kit* and *FcεRI*, suggesting a putative role of mast cells in the different histamine responses. As the mast cell counts obtained by quantitative immunohistochemistry were not different between the experimental groups, altered gene expression levels of the mast cells can be assumed. C-Kit as a cytokine receptor and FcεRI as a high-affinity receptor for immunoglobulin E can modulate the biological behaviour of mast cells, which would require an in depth-characterization. Mast cells seem to be of major relevance with regard to intestinal histamine effects, as histamine release from mast cells is considered to be an important mechanism in inflammatory or allergic processes leading to abdominal pain, discomfort and hypersecretion [42]. The information of dietary effects on the tissue mast cell levels, their degranulation and the resulting histamine levels is scarce, but dietary fat or physical structure of the diet have been identified as possible factors [43,44]. 

Proceeding from increased luminal histamine levels of not defined origin, the present data suggest a counter-regulatory adaptation of the colonic tissue towards overall increased histamine concentrations in the high fCP groups. The activity of the histamine-degrading enzymes DAO and HMT was enhanced in these groups. The increased activity of HMT coincided with an upregulation of HMT gene transcription, while increased activity of DAO appeared to have a purely posttranscriptional nature. Both enzymes would limit the bioavailability of histamine upon entry from the lumen or release from mast cells [22,23] with implications for the effects of histamine on the colonic tissue.

High protein diets are often associated with changes in faecal consistency, possibly due to metabolites such as histamine or PGE_2_. Although they can induce the secretory response of the colonic epithelium in the pig, these effects were counter-regulated with diets containing high levels of fermentable protein. For histamine, this effect seemed to be mainly facilitated through a reduced efficacy of histamine to stimulate chloride secretion via the cAMP/CFTR pathway and an increased epithelial catabolism of histamine due to higher mRNA abundance of *HMT* and higher activities of DAO and HMT in the colonic tissue. In addition, these effects were independent of the inclusion of fCHO in the diet. 

## Supporting Information

Table S1 **Ingredients and nutrient composition of the four experimental diets containing high or low concentrations of fermentable carbohydrates (fCHO) or fermentable protein (fCP).**
(DOC)

Table S2 **List of primers used in this study.**
(DOC)

Table S3 **Baseline of colonic tissue short circuit current (Isc; µA/cm^2^) and tissue conductance (Gt; mS/cm^2^) 3 min before the application of carbachol, PGE2, histamine and NaHS of piglets fed diets containing low or high concentration of fermentable protein (fCP) or fermentable carbohydrates (fCHO).**
(DOC)
