# Supplementary material for: Diets High in Heat-Treated Soybean Meal Reduce the Histamine-Induced Epithelial Response in the Colon of Weaned Piglets and Increase Epithelial Catabolism of Histamine
Source: PLoS One. 2013 Nov 19;8(11):e80612. doi: 10.1371/journal.pone.0080612 (PMC3833947; doi:10.1371/journal.pone.0080612)
Supplement: Table S1 — Ingredients and nutrient composition of the four experimental diets containing high or low concentrations of fermentable carbohydrates (fCHO) or fermentable protein (fCP). (DOC) [file pone.0080612.s001.doc]

| **Table S1.** Ingredients and nutrient composition of the four experimental diets containing high or low concentrations of fermentable carbohydrates (fCHO) or fermentable protein (fCP) | | | | | |
| --- | --- | --- | --- | --- | --- |
|  | low fCP | |  | high fCP | |
|  | low  fCHO | high  fCHO |  | low  fCHO | high  fCHO |
| Ingredients | g/kg as fed | | | | |
| Corn | 402 | 261 |  | 563 | 420 |
| Wheat | 400 | 400 |  | 50 | 50 |
| Soybean meal (44% CP) | 120 | 120 |  | 120 | 120 |
| Heat-treated soybean meal^1^ | - | - |  | 200 | 200 |
| Wheat bran | - | 80 |  | - | 80 |
| Sugar beet pulp | - | 50 |  | - | 50 |
| Monocalcium phosphate | 20 | 16 |  | 20 | 18 |
| Limestone | 19 | 19 |  | 18 | 18 |
| Soybean oil | 10 | 25 |  | 10 | 25 |
| Mineral/Vitamin premix^2^ | 15 | 15 |  | 15 | 15 |
| Salt | 1.5 | 1.5 |  | 1.5 | 1.5 |
| L-Lysine HCl | 6.0 | 5.8 |  | 0.2 | - |
| DL-Methionine | 1.5 | 1.5 |  | 0.5 | 0.5 |
| L-Threonine | 2.0 | 2.0 |  | - | - |
| L-Tryptophan | 1.0 | 1.0 |  | - | - |
| Titanium oxide | 2.0 | 2.0 |  | 2.0 | 2.0 |
|  |  |  |  |  |  |
| Nutrient composition | g/kg dry matter | | | | |
| Crude protein | 145 | 148 |  | 198 | 201 |
| SID^3^ Lysine | 9.9 | 9.9 |  | 9.8 | 9.7 |
| SID Methionine | 3.4 | 3.4 |  | 3.3 | 3.2 |
| SID Threonine | 6.3 | 6.2 |  | 6.9 | 6.3 |
| SID Tryptophan | 2.4 | 2.5 |  | 2.2 | 2.3 |
| Calcium | 11.2 | 11.1 |  | 11.5 | 11.7 |
| Phosphorus | 7.5 | 7.4 |  | 8.0 | 8.3 |
| Sodium | 2.5 | 2.7 |  | 2.6 | 2.7 |
| Total dietary fibre | 145 | 166 |  | 145 | 180 |
| Insoluble dietary fibre | 101 | 130 |  | 110 | 151 |
| Soluble dietary fibre | 44 | 36 |  | 35 | 29 |
| Starch | 488 | 413 |  | 392 | 315 |
| Metabolisable energy (*MJ/kg)* | 13.0 | 12.7 |  | 13.0 | 12.7 |

^1^ Steam autoclaved at 124 ˚C for 20 min

^2^ Mineral and Vitamin Premix (Spezialfutter Neuruppin Ltd., Neuruppin, Germany), containing per kg dry matter: 130 g sodium (as sodium chloride), 55 g magnesium (as magnesium oxide), 210 mg retinol, 3000 µg cholecalciferol, 8000 mg d,l-α-tocopherol, 300 mg menadione, 250 mg thiamin, 250 mg riboflavin, 400 mg pyridoxine, 2000 µg cyanocobalamin, 2500 nicotinic acid, 100 mg folic acid, 25,000 µg biotin, 1000 mg pantothenic acid, 80,000 mg choline chloride, 5000 mg iron (as iron-(II)-carbonate), 1000 mg copper (as copper-(II)- sulphate), 5000 mg zinc (as zinc oxide), 6000 mg manganese (as manganese-(II)-oxide), 45 mg iodine (as calcium-iodate), 35 mg selenium (as sodium-selenite)

^3^ SID = Standardized Ileal Digestible
