# Supplementary material for: Diets High in Heat-Treated Soybean Meal Reduce the Histamine-Induced Epithelial Response in the Colon of Weaned Piglets and Increase Epithelial Catabolism of Histamine
Source: PLoS One. 2013 Nov 19;8(11):e80612. doi: 10.1371/journal.pone.0080612 (PMC3833947; doi:10.1371/journal.pone.0080612)
Supplement: Table S2 — List of primers used in this study. (DOC) [file pone.0080612.s002.doc]

**Table S2.** List of primers used in this study

| Target | Sequences of primers (5` to 3`) | A_T_^1^ |
| --- | --- | --- |
| 18S | TCCGACTTTCGTTCTTGATTAATG | 60 |
|  | TGGACCGGCGCAAGAC |  |
| Beta actin | AGAGCGCAAGTACTCCGTGT | 60 |
|  | ACATCTGCTGGAAGGTGGAC |  |
| 60S ribosomal protein L19 (RPL19) | GCTTGCCTCCAGTGTCCTC | 60 |
|  | GCGTTGGCGATTTCATTAG |  |
| Hypoxanthine phosphoribosyltransferase 1 (HRPT1) | GGACTGAACGGCTTGCTC | 60 |
|  | CAGAGGGCTACGATGTGATG |  |
| Ameloride binding protein (*DAO*) | GCCTGAAGCCGCCCCCTTTT | 60 |
|  | TGTGGGGGAACCTCGGGCTT |  |
| Histamine *N*-Methyl Transferase (*HMT*) | GGAGCTTGTTTTCTGACCACGGCA | 60 |
|  | TCCTGCATGCACTGGTGTTCCG |  |
| Stem cell growth factor receptor (*c-Kit*) | AGCCGACTAGCCTCAGAATGGC | 60 |
|  | CCTGCCGGAGCCATGCAGTAA |  |
| Fc-epsilon receptor I (*FcεRI*) | GTCGACTCAAGCTCCAGGTGCG | 60 |
|  | GCCCGTGTAAACGCCGTCTGA |  |
| Cystic fibrosis transmembrane conductance regulator (*CFTR*) | CCTTGGCGCACTTCGTGTGGA | 60 |
|  | AAGGCGGAGGCCTGCAACAA |  |

^1^A_T_, Annealing temperature in °C
