# Supplementary material for: Diets High in Heat-Treated Soybean Meal Reduce the Histamine-Induced Epithelial Response in the Colon of Weaned Piglets and Increase Epithelial Catabolism of Histamine
Source: PLoS One. 2013 Nov 19;8(11):e80612. doi: 10.1371/journal.pone.0080612 (PMC3833947; doi:10.1371/journal.pone.0080612)
Supplement: Table S3 — Baseline of colonic tissue short circuit current (Isc; µA/cm2) and tissue conductance (Gt; mS/cm2) 3 min before the application of carbachol, PGE2, histamine and NaHS of piglets fed diets containing low or high concentration of fermentable protein (fCP) or fermentable carbohydrates (fCHO). (DOC) [file pone.0080612.s003.doc]

**Table S3.** Baseline of colonic tissue short circuit current (Isc; µA/cm^2^) and tissue conductance (Gt; mS/cm^2^) 3 min before the application of carbachol, PGE_2_, histamine and NaHS of piglets fed diets containing low or high concentration of fermentable protein (fCP) or fermentable carbohydrates (fCHO)

|  | low fCP | | | | high fCP | | | | *P* values | | |
| --- | --- | --- | --- | --- | --- | --- | --- | --- | --- | --- | --- |
|  | low fCHO | | high fCHO | | low fCHO | | high fCHO | | fCP | fCHO | fCP x fCHO |
| Isc (µA/cm^2^) | Mean | SE | Mean | SE | Mean | SE | Mean | SE |  |  |  |
| Carbachol | -20.0 | 10 | -14.4 | 3.8 | -20.6 | 3.6 | -26.2 | 4.1 | 0.216 | 0.995 | 0.261 |
| PGE_2_ | -26.4 | 6.7 | -18.1 | 2.7 | -15.5 | 3.5 | -23.0 | 2.6 | 0.383 | 0.908 | 0.032 |
| Histamine | -13.6 | 10 | -14.9 | 3.8 | -18.4 | 2.2 | -24.9 | 6.0 | 0.159 | 0.463 | 0.626 |
| NaHS | -15.8 | 3.5 | -12.9 | 2.3 | -15.8 | 4.4 | -22.1 | 3.5 | 0.132 | 0.573 | 0.137 |
| Gt (mS/cm^2^) |  |  |  |  |  |  |  |  |  |  |  |
| Carbachol | 16.2 | 5.1 | 14.8 | 1.3 | 13.7 | 1.4 | 16.2 | 2.3 | 0.820 | 0.820 | 0.420 |
| PGE_2_ | 15.7 | 3.1 | 13.9 | 1.3 | 12.3 | 1.1 | 15.0 | 2.6 | 0.731 | 0.869 | 0.611 |
| Histamine | 14.3 | 3.4 | 13.9 | 1.3 | 12.3 | 1.1 | 15.0 | 2.6 | 0.831 | 0.562 | 0.424 |
| NaHS | 14.1 | 2.0 | 14.1 | 1.1 | 14.0 | 1.9 | 12.8 | 1.4 | 0.640 | 0.652 | 0.646 |

PGE_2_, prostaglandin E_2_; NaHS, sodium hydrogen sulphite; fCP, fermentable crude protein; fCHO fermentable carbohydrates
